# Otitis in a cat associated with *Corynebacterium provencense*

**DOI:** 10.1186/s12917-018-1526-9

**Published:** 2018-06-25

**Authors:** Sonja Kittl, Isabelle Brodard, Lorenz Rychener, Jörg Jores, Petra Roosje, Stefanie Gobeli Brawand

**Affiliations:** 1 Institute of Veterinary Bacteriology, Vetsuisse Faculty, University of Bern, Laenggassstrasse 122, CH-3001 Bern, Switzerland; 2 Division of Clinical Dermatology, Department of Clinical Veterinary Medicine, Vetsuisse Faculty, University of Bern, Bremgartenstrasse 109a, CH-3001 Bern, Switzerland; 3 DermFocus, Vetsuisse Faculty, University of Bern, Bern, Switzerland

**Keywords:** Feline otitis, *Corynebacterium*, Neglected pathogen

## Abstract

**Background:**

The role of corynebacteria in canine and feline otitis has not been investigated in detail; however, members of this genus are increasingly recognized as pathogens of otitis in both human and veterinary medicine.

**Case presentation:**

Here we report the first case of feline otitis associated with the recently described species *Corynebacterium provencense*. A seven-month old cat presented with a head tilt and ataxia was diagnosed with peripheral vestibular syndrome associated with an otitis media/interna. This took place 6 weeks after resection of a polyp, having initially shown a full recovery with topical ofloxacin and glucocorticoid treatment. Bacteriology of an ear swab yielded a pure culture of corynebacteria, which could not be identified at the species level using routine methods. However, the 16S rRNA gene sequence was 100% identical to the recently published novel corynebacterium species, *Corynebacterium provencense*. Whole genome sequencing of the cat isolate and calculation of average nucleotide identity (99.1%) confirmed this finding. The cat isolate was found to contain additional presumptive iron acquisition genes that are likely to encode virulence factors. Furthermore, the strain tested resistant to clindamycin, penicillin and ciprofloxacin. The cat was subsequently treated with chloramphenicol, which lead to clinical improvement.

**Conclusion:**

Corynebacteria from otitis cases are not routinely identified at the species level and not tested for antimicrobial susceptibility in veterinary laboratories, as they are not considered major pathogens. This may lead to underreporting of this genus or animals being treated with inappropriate antimicrobials since corynebacteria are often resistant to multiple drugs.

**Electronic supplementary material:**

The online version of this article (10.1186/s12917-018-1526-9) contains supplementary material, which is available to authorized users.

## Background

The genus *Corynebacterium* consists of 128 species [1], with a large number pathogenic to humans and animals [2]. The main animal pathogens include *C. pseudotuberculosis*, *C. kutscheri*, the *C. renale* group, *C. bovis* and *C. ulcerans* while *C. diphtheriae* is considered the main human pathogen [3]. Many other *Corynebacterium* spp. have been isolated from opportunistic infection sites in humans and animals, sometimes also with a zoonotic origin [2]. Besides the diphtheria toxin, which is the major virulence factor in *C. diphtheriae* (also found in other species like *C. ulcerans*) [4], and phospholipase D, a virulence factor of *C. pseudotuberculosis* and *C. ulcerans* [5], relatively little is known about virulence traits in the genus *Corynebacterium*.

Recently human and veterinary diagnostic laboratories have begun to investigate the role of *Corynebacterium* spp. as causative agents for otitis with a study [6] finding *Corynebacterium* spp. to be present in 33% of human patients with otologic infections. These cases were often resistant to fluoroquinolones, an antibiotic frequently used as a first-line in treatment [6]. In dogs, *Corynebacterium* spp*.* are also increasingly recognized as otitis-associated pathogens, however, in most cases they are isolated together with other pathogens [7, 8]. There are even fewer publications describing corynebacteria-associated otitis in cats than in dogs. Henneveld et al. described four cats with otitis externa/media from which corynebacteria were isolated, albeit in association with other bacterial species [7]. Two of these cats also suffered from an aural polyp.

In addition to scant information linking corynebacteria and otitis, knowledge is also limited concerning the microbiome of the healthy feline ear canal. Kose et al. investigated the flora of the normal tympanic bulla in cats but did not detect any corynebacteria, although the sample size was very small (five animals) [9].

Thus, further research is needed to elucidate the role of pathogenic bacteria in feline otitis. In this study, we present a case of feline otitis media and interna from which the recently described species *Corynebacterium provencense* was isolated in pure culture.

## Case presentation

A 7-month-old male, neutered Maine Coon cat was presented to the Small Animal Teaching Hospital at the University of Bern with acute neurological signs consistent with unilateral otitis media/interna. Six weeks earlier an inflammatory aural polyp had been removed by traction and flushing of the ear canal. The cat had fully recovered after three weeks of treatment with oral and topical glucocorticoids and topical ofloxacin (Floxal, Bausch & Lomb Swiss AG). Otoscopic and cytologic examinations revealed brown-colored fluid in the external canal with numerous extra- and intracellular rod-shaped bacteria and neutrophils. A deep ear swab was submitted for culture and subsequent antimicrobial susceptibility testing.

The ear swab was cultured on sheep blood agar at 37 °C for 2 days, yielding a pure culture of small white colonies (strain number 17KM38). Gram staining showed Gram-positive, polymorphic rods and the bacteria were catalase positive. Thus, the bacteria were classified as belonging to the genus *Corynebacterium*, however species identification was not possible with either Maldi-Tof MS (MALDI Biotyper, Bruker using the in-house database and MBT 6903 MSP Library, Bruker) or VITEK® 2 Compact (Biomérieux) (cards GP and CBC). Therefore, the 16S rRNA gene was amplified and Sanger sequenced using universal primers [10]. Sequence analysis and sequence comparison using the BLAST program (NCBI, ‘rRNA_typestrains/prokaryotic_16S_ribosomal_RNA’ database) revealed 98.6% identity to *Corynebacterium variabile* (NR_025314.1), 98.0% to *Corynebacterium terpenotabidum* (NR_121699.1) and 97.8% to *Corynebacterium glyciniphilum* (NR_121782.1), thus the strain 17KM38 could not be assigned to any species present in the database. However, the 16S rRNA sequence showed a 100% identity with the whole genome shotgun sequence of the recently described *Corynebacterium provencense* SN15 (GenBank accession no.: NZ_LT160593.1) isolated from faeces of an obesity patient [11].

In order to select the appropriate antimicrobial therapy, the isolate was tested for antimicrobial resistance using broth microdilution (Sensititre EUST, Thermo Fisher Scientific) initially following in-house guidelines. Minimum inhibitory concentration (MIC) testing was however repeated according to the most recent EUCAST (European Committee on Antimicrobial Susceptibility Testing) guidelines [12] using *Streptococcus pneumonia*e ATCC 49619 as a quality control. Briefly, the bacteria were grown on sheep blood agar at 37 °C for 24 h. They were then suspended in Mueller-Hinton broth with 5% lysed horse blood (Thermo Fisher Scientific) and 20 mg/l β-NAD to a concentration of 5 × 10^5^ CFU/ml and used for plate inoculation. Our strain tested resistant to clindamycin, penicillin and ciprofloxacin but susceptible to tetracycline, gentamicin and vancomycin. In addition, high MIC values were found for cefoxitin (8 mg/l), mupirocin (≥256 mg/l) and trimethoprim (≥32 mg/l) while the MIC for chloramphenicol was low (≤4 mg/l) (Table 1).

**Table 1:** Minimal inhibitory concentrations for 17KM38, interpretation according to EUCAST clinical breakpoints where available

Antimicrobial	MIC (mg/l)	Interpretation
Clindamycin	2	R
Tetracycline	1	S
Rifampin	0.25	I
Streptomycin	≤4	–
Fusidate	1	–
Penicillin	1	R
Chloramphanicol	≤4	–
Kanamycin	≤4	–
Tiamulin	≥4	–
Quinupristin/dalfopristin	1	–
Vancomycin	≤1	S
Gentamicin	≤1	S
Trimethoprim	≥32	–
Erythromycin	≤0.25	–
Ciprofloxacin	≥8	R
Cefoxitin	8	–
Linezolid	≤1	–
Mupirocin	≥256	–
Sulfamethoxazole	≤64	–

**Fig. 1:**
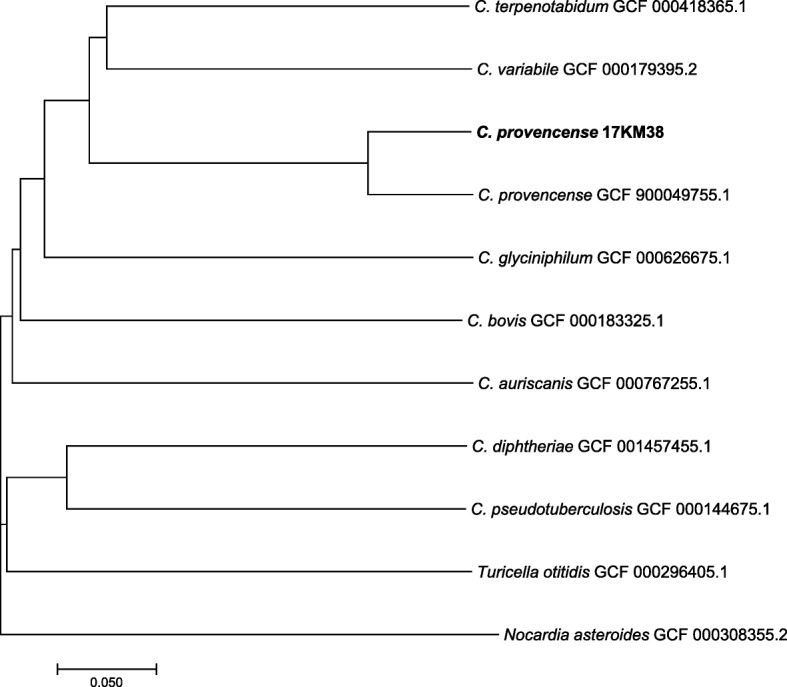
Phylogenetic tree based on the proteome, showing 17KM38 in relation to other corynebacteria. The tree was constructed using CVTree with K = 6. *Nocardia asteroides* was used as outgroup. The scale bar indicates normalized distance between composition vectors

To better characterize the strain, whole genome sequencing was performed using the PacBio method. The strain was grown on sheep blood agar at 37 °C for 24 h and the harvested cells were used to extract gDNA according to a protocol published earlier [13]. Sequencing was performed by the Lausanne Genomic Technologies Facility (University of Lausanne, Switzerland). The genome was assembled from PacBio reads using Canu 1.4 [14]. A single contig was built after assembly of the obtained reads, and circularized using amos 3.1.0 [15]. Subsequently the genome was annotated using Prokka 1.12 [16] and submitted to GenBank (Accession No. CP024988). The genome of 17KM38 has a size of 3.11 Mb and 2682 coding sequences were detected. Of these, 862 out of 2682 were annotated as hypothetical proteins. In order to confirm that 17KM38 belongs to the same species as SN15 and is indeed distinct from its closest relatives, average nucleotide identity (ANI) was calculated according to Goris et al. [17] using an online tool (ANI calculator) developed by Rodriguez-R and Konstantinidis [18] with default settings. The ANI of 17KM38 was found to be 81.5% with *C. variabile* (NC_015859.1), 81.0% with *C. terpenotabidum* (NC_021663.1), 79.1% with *C. glyciniphilum* (CP006842.1) and 99.1% with SN15 (FIZC01). Since the recommended cutoff point for species delineation is 95% ANI [17], these results confirm that SN15 [11] and 17KM38 indeed belong to the same species which is distinct from related species. Furthermore, a phylogenetic tree was constructed from the theoretical proteome according to Qi et al. [19] applying the online tool CVTree [20] (Fig. 1).

**Fig. 2:**
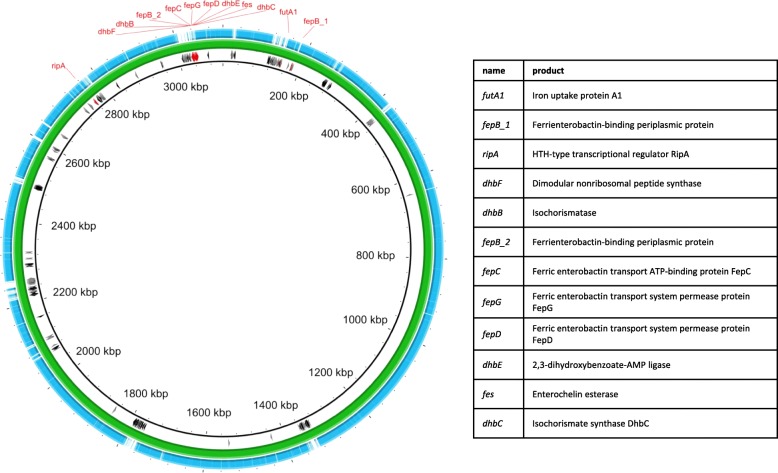
Comparison of 17KM38 (green) with SN15 *Corynebacterium provencense* (blue). CDS in gap regions are shown. Hypothetical proteins are shown in grey, proteins presumptively related to iron acquisition are shown in red. The image was constructed with BRIG (BLAST Ring Image Generator v0.95)

**Fig. 3:**
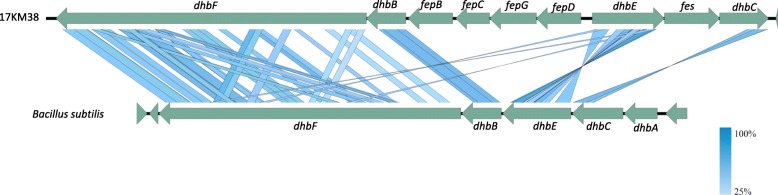
Comparison of the presumptive siderophore synthesis genomic region in 17KM38 and the *dhb* genes containing region in Bacillus subtilis (NC_000964 region: 3280000–3,293,000). The graph was generated using Easyfig 2.2.2 [23]. Comparisons were performed using tblastx, min. Length 50, max. e value 0.001

The genome of 17KM38 was compared to the genome of the strain SN15 using mauve [21]. Gap regions were extracted and annotated resulting in 280 coding sequences (CDS), 113 of which could be assigned a putative function (Additional file 1). Interestingly, 12 CDS unique to 17KM38 are related to iron acquisition (Fig. 2), which is important for bacterial survival in the host and thus for virulence [22]. Most interesting is an approximately 17kbp region containing nine genes for siderophore synthesis and transport organized in two presumptive operons (Fig. 3) [23]. Genes *dhbBCEF* are related to the corresponding genes in *Bacillus subtilis* (between 36 and 52% identity in blastx) where they form the biosynthetic pathway for the catecholic siderophore bacillibactin [24]. The whole gene cluster has similarity to the enterobactin gene cluster in *E. coli* which also includes *fep* genes and the *fes* gene [25]. A gene corresponding to *dhbA*/*entA* is missing from this cluster in 17KM38, but is located elsewhere on the genome (1,026,704 - > 1,027,528). *C. variabile*, the closest relative to *C. provencense*, also possesses a repertoire of iron acquisition genes, since its habitat is the iron-restricted environment found in cheese [26]. Interestingly the genome of *C. variabile* also encodes a pathway similar to that for bacillibactin. However, the *dhbBCEF* genes in 17KM38 are no closer related to those in *C. variabile* than those in *B. subtilis*.

In terms of virulence factors 17KM38 encodes a candidate adhesin (Csp1_16740) related to (82% query cover, 51% identity in blastx) a cell wall-associated hydrolase of *C. resistens* (encoded by *cwlH*) [27]. The exact function of this protein is unknown, however knockout of the homologous gene DIP1621 in *C. diphtheriae* led to reduced adherence to epithelial cells [28]. This gene is also present in strain SN15.

As mentioned above, 17KM38 showed resistance to several antibiotics. Quinolone resistance in corynebacteria has been described to rely on mutations in the quinolone resistance determining region (QRDR) of *gyrA* leading to changes in the SAIYD (aa 87–91 in *C. glutamicum*) motif of susceptible strains [29]. Mutations of Ser-87 to Arg in *C. amycolatum* and to Val in *C. striatum* have been shown to confer quinolone resistance [29]. 17KM38 showed a mutation in this region changing Ser to Ala, which could explain the quinolone resistance. A mechanism for resistance to ß-lactams in corynebacteria has been demonstrated for *C. jeikeium* where acquisition of the gene *pbp2C* encoding the low-affinity class B penicillin binding protein 2C was shown to confer resistance [30]. 17KM38 has four genes encoding penicillin binding proteins one of which is related to *pbp2C* (Csp1_14570, 96% query cover, 44% identity), however, if this gene is actually related to penicillin resistance remains to be shown.

Following sensitivity testing results, the cat was treated with oral chloramphenicol *palmitate solution (Cloropal,* Dr. E. Gräub AG) 20 mg/kg twice daily for three weeks and an ear cleaner containing chlorhexidine digluconate (Otodine®, Ufamed AG) twice daily for two weeks. Re-examination after three weeks of therapy revealed that the head tilt was much improved and the cat was no longer ataxic and cytology of the ear canal did not show any bacteria or neutrophils.

## Discussion and conclusion

Ear infections with corynebacteria in dogs and cats are rather rare; however, they are of concern to clinicians as they can be difficult to treat due to antibiotic resistance. This has also been described in human medicine where fluoroquinolones are normally used as a first-line therapy for otitis media [6]. A retrospective study by Crowson et al. found 58% of *Corynebacterium sp*. isolates from otitis patients were resistant to ciprofloxacin, compared to only 32% of non-*Corynebacterium* isolates [6]. In dogs and cats, quinolones are also frequently used to treat otitis. Aalbaek et al. found US *Corynebacterium* isolates from canine otitis to show a high percentage of enrofloxacin resistance when compared to Danish isolates [8]. In the case described here, the animal had also initially received topical quinolone therapy from the referring veterinarian before removal of the ear polyp. As cultures were not performed prior to this treatment, the nature of the initial infectious agent is unknown. Possibly a different bacterium caused the first infection, and the *Corynebacterium provencense* was able to proliferate under therapy. Alternatively, as quinolone resistance is presumably caused by a point mutation, the bacteria may have developed resistance under therapy as has been described for other species [31].

As mentioned before, not much is known about virulence factors of corynebacteria, however, iron acquisition systems are known to contribute to virulence in many pathogens [22]. Interestingly, the strain in this study contained a genomic region coding for a siderophore synthesis pathway that was not present in the human *C. provencense* strain and therefore might contribute to its virulence. As corynebacteria from ear infections are not routinely identified to species level in many laboratories, cases with this species may have been previously underreported. It is therefore desirable to pay more attention to corynebacterial ear infections in dogs and cats with importance placed on resistance testing as is increasingly done in human medicine. Consequently, this will improve the treatment success rate for otitis in veterinary medicine.

## Additional file

Additional file 1: Annotations of Gap regions comparing 17KM38 and SN15. The genome sequences of 17KM38 and SN15 were compared with mauve and gap regions extracted. Annotations of these regions are shown here (XLSX 21 kb).

## Abbreviations

ANI: Average Nucleotide Identity
CDS: Coding Sequence
EUCAST: European Committee on Antimicrobial Susceptibility Testing
MIC: Minimum Inhibitory Concentration

## References

[1] LPSN List of prokaryotic names with standing in nomenclature http://www.bacterio.net/corynebacterium.html Accessed 19 June 2017. 10.1093/nar/gkt1111 PMC3965054 24243842

[2] Bernard K (2012). The genus corynebacterium and other medically relevant coryneform-like bacteria. J Clin Microbiol.

[3] Markey BK (2013). Clinical veterinary microbiology.

[4] Meinel DM, Margos G, Konrad R, Krebs S, Blum H, Sing A (2014). Next generation sequencing analysis of nine Corynebacterium ulcerans isolates reveals zoonotic transmission and a novel putative diphtheria toxin-encoding pathogenicity island. Genome Med.

[5] Tauch A, Burkovski A (2015). Molecular armory or niche factors: virulence determinants of Corynebacterium species. FEMS Microbiol Lett.

[6] Crowson MG, Callahan K, Saunders JE (2015). Review of otorrhea microbiology: is there a pathogenic role of corynebacter?. Otol Neurotol.

[7] Henneveld K, Rosychuk RA, Olea-Popelka FJ, Hyatt DR, Zabel S (2012). Corynebacterium spp. in dogs and cats with otitis externa and/or media: a retrospective study. J Am Anim Hosp Assoc.

[8] Aalbaek B, Bemis DA, Schjaerff M, Kania SA, Frank LA, Guardabassi L (2010). Coryneform bacteria associated with canine otitis externa. Vet Microbiol.

[9] Klose TC, MacPhail CM, Schultheiss PC, Rosychuk RA, Hawley JR, Lappin MR (2010). Prevalence of select infectious agents in inflammatory aural and nasopharyngeal polyps from client-owned cats. J Feline Med Surg.

[10] Kuhnert P, Frey J, Lang NP, Mayfield L (2002). Phylogenetic analysis of Prevotella nigrescens, Prevotella intermedia and Porphyromonas gingivalis clinical strains reveals a clear species clustering. Int J Syst Evol Microbiol.

[11] Ndongo S, Andrieu C, Fournier PE, Lagier JC, Raoult D (2017). Actinomyces provencensis' sp. nov., 'Corynebacterium bouchesdurhonense' sp. nov., 'Corynebacterium provencense' sp. nov. and 'Xanthomonas massiliensis' sp. nov., 4 new species isolated from fresh stools of obese French patients. New Microbes New Infect.

[12] Breakpoint tables for interpretation of MICs and zone diameters. Version 8.1, 2018**.** http://www.eucast.org

[13] Pitcher DG, Saunders NA, Owen RJ (1989). Rapid extraction of bacterial genomic DNA with guanidium thiocyanate. LettApplMicrobiol.

[14] Koren S, Walenz BP, Berlin K, Miller JR, Bergman NH, Phillippy AM (2017). Canu: scalable and accurate long-read assembly via adaptive k-mer weighting and repeat separation. Genome Res.

[15] AMOS http://amos.sourceforge.net/wiki/index.php/AMOS Accessed 03 July 2017.

[16] Seemann T (2014). Prokka: rapid prokaryotic genome annotation. Bioinformatics.

[17] Goris J, Konstantinidis KT, Klappenbach JA, Coenye T, Vandamme P, Tiedje JM (2007). DNA-DNA hybridization values and their relationship to whole-genome sequence similarities. Int J Syst Evol Microbiol.

[18] Online tools Environmental Microbial Genomics Laboratory http://enve-omics.ce.gatech.edu/ Accessed 07 June 2017.

[19] Qi J, Wang B, Hao BI (2004). Whole proteome prokaryote phylogeny without sequence alignment: a K-string composition approach. J Mol Evol.

[20] Composition Vector Tree Version 2 http://tlife.fudan.edu.cn/cvtree Accessed 17 July 2017.

[21] Darling AC, Mau B, Blattner FR, Perna NT (2004). Mauve: multiple alignment of conserved genomic sequence with rearrangements. Genome Res.

[22] Chu BC, Garcia-Herrero A, Johanson TH, Krewulak KD, Lau CK, Peacock RS, Slavinskaya Z, Vogel HJ (2010). Siderophore uptake in bacteria and the battle for iron with the host; a bird's eye view. Biometals.

[23] Sullivan MJ, Petty NK, Beatson SA (2011). Easyfig: a genome comparison visualizer. Bioinformatics.

[24] May JJ, Wendrich TM, Marahiel MA (2001). The dhb operon of Bacillus subtilis encodes the biosynthetic template for the catecholic siderophore 2,3-dihydroxybenzoate-glycine-threonine trimeric ester bacillibactin. J Biol Chem.

[25] Crosa JH, Walsh CT (2002). Genetics and assembly line enzymology of siderophore biosynthesis in bacteria. Microbiol Mol Biol Rev.

[26] Schroder J, Maus I, Trost E, Tauch A (2011). Complete genome sequence of Corynebacterium variabile DSM 44702 isolated from the surface of smear-ripened cheeses and insights into cheese ripening and flavor generation. BMC Genomics.

[27] Schroder J, Maus I, Meyer K, Wordemann S, Blom J, Jaenicke S, Schneider J, Trost E, Tauch A (2012). Complete genome sequence, lifestyle, and multi-drug resistance of the human pathogen Corynebacterium resistens DSM 45100 isolated from blood samples of a leukemia patient. BMC Genomics.

[28] Kolodkina V, Denisevich T, Titov L (2011). Identification of Corynebacterium diphtheriae gene involved in adherence to epithelial cells. Infect Genet Evol.

[29] Sierra JM, Martinez-Martinez L, Vazquez F, Giralt E, Vila J (2005). Relationship between mutations in the gyrA gene and quinolone resistance in clinical isolates of Corynebacterium striatum and Corynebacterium amycolatum. Antimicrob Agents Chemother.

[30] Lavollay M, Arthur M, Fourgeaud M, Dubost L, Marie A, Riegel P, Gutmann L, Mainardi JL (2009). The beta-lactam-sensitive D,D-carboxypeptidase activity of Pbp4 controls the L, D and D, D transpeptidation pathways in Corynebacterium jeikeium. Mol Microbiol.

[31] Luo N, Sahin O, Lin J, Michel LO, Zhang Q (2003). In vivo selection of campylobacter isolates with high levels of fluoroquinolone resistance associated with gyrA mutations and the function of the CmeABC efflux pump. Antimicrob Agents Chemother.

